# Mechanochemical interactions in cancer cells: The role of substrate stiffness in cell behavior and drug response

**DOI:** 10.1371/journal.pone.0327874

**Published:** 2026-01-07

**Authors:** Sayed Reza Ramezani, Afsaneh Mojra, Mohammad Tafazzoli-Shadpour

**Affiliations:** 1 Department of Mechanical Engineering, K. N. Toosi University of Technology, Tehran, Iran; 2 Department of Biomedical Engineering, Amirkabir University of Technology, Tehran, Iran; Advanced Materials Technology Research Institute, National Research Centre, EGYPT

## Abstract

Cancer cells adhere to the extracellular matrix, where they sense and respond to variations in substrate stiffness, influencing their proliferation and invasive potential. Numerous studies have examined the biological activities of cells in relation to mechanical forces; however, research addressing the combined effects of mechanical and chemical interactions on cancer cell behavior across different metastatic stages remains limited. Moreover, the influence of chemotherapeutic drugs in the context of specific cellular characteristics remains underexplored. Therefore, in this study, synthetic polyacrylamide gels with varying elastic moduli were utilized to effectively mimic the diversity of host tissue environments for prostate cancer cells. Additionally, cellular behavior of prostate cancer cells with differing metastatic potential—low (LNCaP), medium (DU145), and high (PC3)—was evaluated in response to anticancer drugs. Ultimately, effects of drug treatment were comprehensively examined using Docetaxel, Bicalutamide, and Abiraterone Acetate, which target distinct cellular components and activate diverse signaling pathways. The assessments were based on the analysis of actin filament content and organization, size of nucleus, and cellular elastic modulus. The results revealed that a soft substrate improves the medication efficacy, resulting in an enhanced cell death rate of 40–60% compared to 20–30% on a stiff substrate. Cells cultured on soft substrates exhibited lower phalloidin content (8–16%) compared to those on stiff substrates (18–32%). Additionally, drug treatments influenced cell mechanics, with Docetaxel reducing the elastic modulus, while Bicalutamide induced an increase. Based on these findings, a treatment strategy aimed at enhancing therapeutic efficacy can be proposed.

## Introduction

Prostate cancer (PCa) is the second most common cancer in men worldwide [1–3]. It shows significant molecular variability within and between tumor foci in individuals as well as across foci in different patients [4]. In contrast to most cancers, PCa exhibits few identifiable genetic drivers [5,6], which makes it difficult to determine the mechanisms guiding its initiation and spread [7]. The migration of cancer cells involves complex functional changes which contribute to their spread, invasion, and metastasis. The first functional change involves dynamic rearrangement of actin cytoskeleton, which changes the cell shape and the membrane structure that connect the cytoskeleton to the extracellular matrix (ECM) [8–10]. By signaling the actin cytoskeleton, sex steroids accelerate the migration and invasion of cancer cells. The rapid activation of moesin, an actin-binding protein associated with the integrin and ECM, facilitates these effects. Actin fibers depolymerize and reassemble toward the cell membrane periphery in response to moesin activation, which results in the creation of cortical actin complexes and specific membrane structures that are essential for cellular motility. Recent findings have highlighted the functional role of ECM proteins in aggressive cancers [11–13]. By connecting to the ECM, integrins transduce signals via integrin-mediated signaling pathways that pass through ECM proteins and affect the mechanical properties of cancer cells [14–19]. Collagen hydrogels, fibrin, alginate, and agarose are used to mimic the ECM properties. The elastic modulus of ECM plays a key role in cancer cell behavior and how mechanical forces are transmitted [20]. In this regard, Tilghman et al. [21] investigated the effect of ECM’s elastic modulus on cancer cell behavior. In this study, cancer cells were classified based on their growth response to substrate rigidity, identifying rigidity-dependent and rigidity-independent phenotypes. Softer substrates induced morphological changes, reduced motility, and increased E-cadherin expression while downregulating slug expression. Additionally, it was confirmed that cancer cell adaptability to soft environments correlate with ECM responsiveness, emphasizing the role of mechanical cues in tumor progression. Mielnicka et al. [22] examined how various elastic substrates influenced the behavior of WC256 Walker carcinosarcoma cells. The study revealed that more rigid substrates promote cell proliferation and invasion, whereas less rigid substrates lead to increased cell death. The outcomes implied that the mechanical characteristics of the tumor microenvironment have the potential to impact the proliferation and advancement of cancer cells. Jeong et al. [23] examined the effect of varying graphene oxide (GO) concentrations on silicon substrates for cell adhesion and growth. Their findings demonstrated that an optimal GO concentration enhances both cell adhesion and proliferation. It was concluded that GO-coated surfaces have potential for biomedical applications, particularly in tissue engineering. However, modifying their elastic modulus without affecting biological characteristics has remained challenging [24–30]. This issue is associated with undesirable reactions due to cell binding to specific receptors and changes in polymer concentration [28–30]. Consequently, research has shifted to synthetic polyacrylamide (PAAM) gels, which are anti-adhesive and biologically inert, allowing precise control of elastic modulus and independent regulation of biological features [20,31–33]. The elastic and isotropic properties of PAAM gels facilitate easy calculation of traction forces, making them widely used in studies of cell-substrate interactions [24–35].

**Fig 1:**
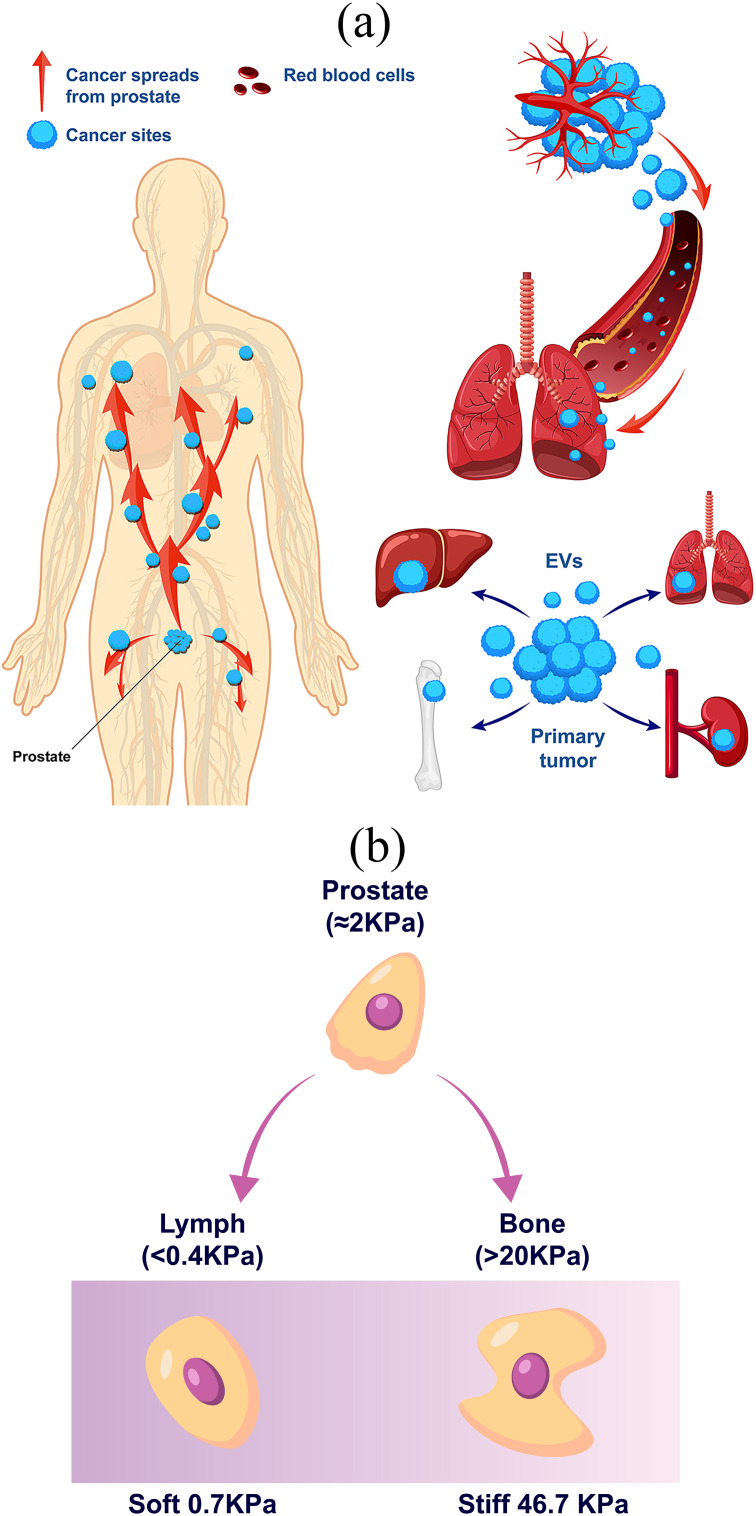
Prostate cancer cells metastasize to other tissues; (a) The spread of prostate cancer cells associated with the elastic modulus sensed by cancer cells, (b) different metastatic locations represented by different substrate elastic moduli.

Studies have also demonstrated that ECM stiffness plays a significant role in cellular drug resistance, affecting how cancer cells respond to treatments [36–38]. Notably, Kalli et al. [36] demonstrated that mechanical forces within the tumor microenvironment—beyond just matrix stiffness—significantly influence cancer drug resistance. They emphasized how force-driven signaling pathways support tumor survival and enable therapeutic evasion. Their findings suggested that disrupting these mechanotransduction mechanisms could open new avenues for overcoming drug resistance and enhancing treatment effectiveness. In drug efficacy screening assays, drug diffusion within the matrix network is a critical factor. Certain substrates exhibit high affinity for small molecules, making them potential carriers for controlled drug release. However, excessive binding between drugs and the matrix can negatively impact the accuracy of drug efficacy studies conducted in cell cultures [39,40]. Studies have revealed that HMGA1 undergoes phosphorylation by the CK2 substrate, enhancing its functionality and contributing to chemotherapy resistance [39,40]. This suggests that the targeted inhibition of HMGA1 and CK2 could provide an effective strategy to overcome drug resistance in lung cancer. Additionally, ensuring adequate drug access to cells requires appropriate diffusion properties within the matrix [39]. Also, evaluation of cancer cell mechanical properties using atomic force microscopy (AFM) has revealed a close relation between changes in cell elastic modulus and cell cytoskeleton, particularly actin filaments [41–52]. However, only a handful of studies have examined the biomechanical alterations in malignant cells exposed to microtubule-targeting medications [48–50,53,54]. As shown in Fig 1, prostate cancer cells exhibit the ability to migrate to various host tissues with different ECM elastic moduli, influencing their behavior and progression. Our literature review suggests that the combined effects of mechanical and chemical interactions on the biological behavior and mechanical properties of cancer cells with varying metastatic potential have been insufficiently explored. Furthermore, the impact of chemotherapeutic drugs in relation to cancer cell characteristics remains underexplored. To account for host tissue diversity, this study utilizes PAAM gel substrates with varying elastic moduli—stiff, medium, and soft—for cancer cell analysis. Additionally, the cellular behavior of prostate cancer cells with different metastatic potentials, including low (LNCaP), medium (DU145), and high (PC3), is assessed in response to various anticancer drugs. Finally, the effects of these medications are thoroughly investigated, focusing on drugs that target distinct cellular components and activate diverse signaling pathways, including Docetaxel, Bicalutamide, and Abiraterone Acetate. The rest of the paper is structured as follows. Section two outlines the methodology, detailing the preparation of cells and drugs as well as the procedures used for evaluation. Section three presents and discusses the results. The findings are concluded in the last section.

## Materials and methods

Three different anticancer medications are used to treat prostate cancer cells with high, mediate and low metastatic potentials and IC50s are calculated using the MTT (3-(4,5-Dimethylthiazol-2-yl)-2,5-diphenyltetrazolium bromide) assay. All tests and analyses are conducted on three prostate cancer cell lines, each representing a different level of metastasis: LNCaP (low), DU145 (medium), and PC3 (high). Cells are cultured on three substrates of various elastic moduli (stiff, medium, and soft) and treated with three anticancer drugs: Docetaxel, Bicalutamide, and Abiraterone Acetate. All data undergo statistical evaluation to examine the mechanical properties of the cells in relation to their respective lines, comparing treated and untreated groups.

### Manufacturing and preparation of acrylamide substrates

**Fig 2:**
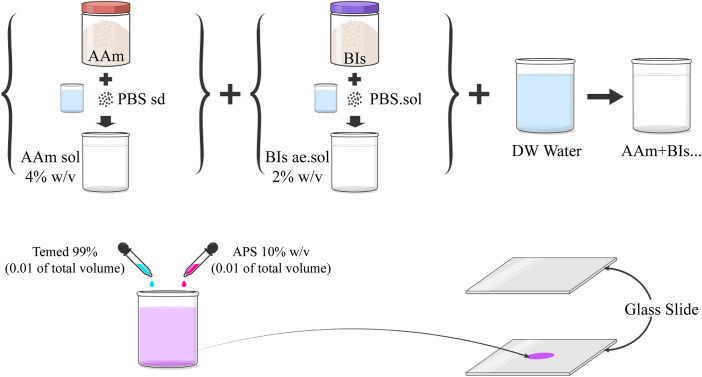
A schematic of the substrate preparation; polyacrylamide gels of varying cross-linking densities are prepared by combining AAm 40% weight/volume, DW, APS, and TEMED of varying concentrations to create cell-culture substrates with different elastic moduli.

In this study, acrylamide-based substrates are fabricated following established protocols to ensure consistency and reproducibility. Acrylamide hydrogels have been extensively used in previous research to examine cell responses to mechanical cues, as their tunable stiffness enables the simulation of different tissue conditions. Additionally, to enhance the physiological relevance of the system and better mimic the adhesive properties of native ECM, fibronectin—a key extracellular matrix protein—is coated onto the substrates. To synthesize PAAM gels, aqueous solutions of Acrylamide monomer (AAm) and N, N′-Methylen bis acrylamide (cross-linker, Bis-acrylamide) are prepared at concentrations of 40% and 2% weight/volume, respectively. Then, various Bis-acrylamide and AAm concentrations are prepared in deionized water (DW). The initiation of the free radical polymerization reaction involves the use of Ammonium Persulfate (APS, 10% weight/volume), catalyzed by N, N, N′, N′-Tetramethylethylenediamine (TEMED 99%). Distinct polyacrylamide gels, each of varying cross-linking (Bis-acrylamide 2% weight/volume) densities, are formulated by combining AAm 40% weight/volume, DW, APS, and TEMED with different concentrations (According to Table 1). After thorough mixing, the gel solution is poured into a well serving as a mold, positioned between two glass slides, and allowed to polymerize for 3 hours at room temperature. Upon achieving complete polymerization, the layers of PAAM gel are meticulously detached from the mold. The gel surface is washed three times with PBS and sterilized with UV light. A schematic of the substrate preparation is shown in Fig 2.

**Table 1:** The elastic modulus of the three substrates (stiff, medium and soft) and concentrations used to polymerize Acrylamide and Bis-Acrylamide; the values are averaged across three samples.

Substrate stiffness migration	Polymerization of Relative Acrylamide, Bis-Acrylamide Concentrations and water			Measured values for modulus of elasticity (kPa)
	Acrylamide from 40% stock solution (ml)	Bis-acrylamide from 2% stock solution (ml)	Water (ml)	
Stiff	2	2.4	5.6	19.325±1.3278
Medium	2	0.24	7.76	1.449±0.1634
Soft	1.25	0.15	8.6	0.7703±0.058

### Substrate young’s modulus

The pressure test under ASTM standards involves applying controlled mechanical stress to acrylamide hydrogel samples to determine their elastic modulus for cell-culture-substrate applications. The substrate elastic modulus is measured using pressure testing, with samples tested in a cylindrical mold measuring 1 inch in diameter and height, applying a strain rate of 20 mm per minute and a 10 N load. Repeatability is assessed through three consecutive tests conducted on each of the nine specimens, following ASTM standards including D695, D638 and D412. Fig S1 in S1 File illustrates the pressure testing procedure on the substrates, the test apparatus and the samples under the test.

**Fig 3:**
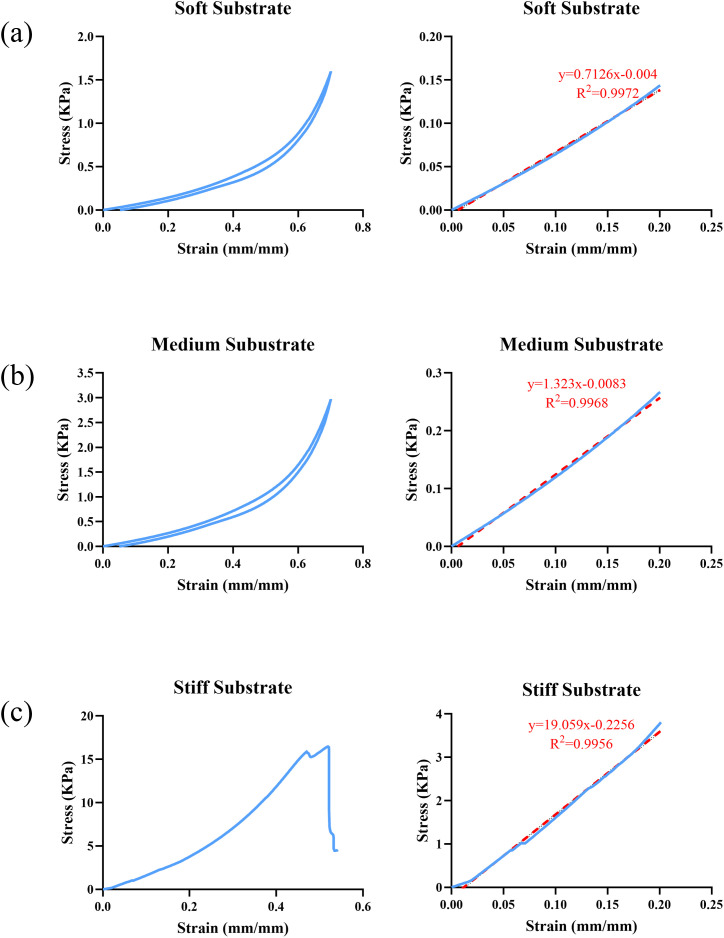
The stress-strain diagrams for the samples in each substrate group, (a) soft substrate, (b) medium substrate, (c) stiff substrate; The diagrams on the left column show the first twenty percent of the total stress versus strain, with the slopes of the passing line represent the elastic moduli.

Fig 3 shows the stress-strain diagrams for a sample of each substrate group. In this figure, the graphs for soft, medium and stiff substrates are shown in Figs 3a‒3c, respectively. Additionally, three replicates are performed for each group. The slope of the line within the elastic region represents the elastic modulus and directly correlates with the substrate’s elastic modulus. For all samples, linear fitting has a very good quality with R-squared values higher than 0.99. Based on the diagrams, the soft samples exhibit smaller slopes compared to the stiff ones, indicating higher ductility under the same applied force. Additionally, exceeding 50% strain causes break-down and fracture of stiff samples, while softer samples resist the strain and complete the loading and unloading stages. Table 1 presents the elastic modulus of samples, and the concentrations used to polymerize them.

### Fibronectin coating and cell seeding

Fibronectin is applied to acrylamide substrates in cell culture to promote cell attachment and facilitate spreading. This extracellular matrix protein promotes interactions between cells and the substrate, mimicking physiological conditions and improving cell viability. One of the primary reasons for using fibronectin is its ability to activate specific signaling pathways, thereby facilitating the differentiation of cells into a particular cell line. Additionally, fibronectin ensures uniform cell adhesion to the substrate across all cells, minimizing variability in adhesion patterns. By facilitating proper cell adhesion, fibronectin coatings enable more accurate studies of cellular response to mechanical and biochemical stimuli in controlled experimental tests. Regarding this, the acrylamide substrates are coated with fibronectin by incubating them with a 2 µg/mL fibronectin solution for one hour at 37°C. Subsequently, the substrates are gently washed with phosphate-buffered saline (PBS) to remove excess fibronectin, ensuring a uniform coating. After fibronectin coating on the substrates, the substrates are ready for cell seeding.

### Cell culture

The National Center for Genetic Resources provides cell lines with low (LNCAP), moderate (DU145), and high (PC3) metastatic potential for the present experimental study. These cell lines are cultured in DMEM medium (Gibco, USA) supplemented with 1% penicillin/streptomycin and 10% fetal bovine serum (FBS; Gibco, USA). The cultures are maintained in an incubator (Memmert, Germany) at 37°C with 5% CO2 until they reach the required cell density. The culture medium is replenished every three days.

### Drug solution preparation

Three dry powder anticancer medicines including Docetaxel, Abiraterone Acetate and Bicalutamide (Sobhan Oncology, Iran) are selected for the present study. The effective concentration (IC50) of each medicine is calculated using the MTT assay. The dry powders are carefully concentrated using DMSO solutions. These solutions are then filtered through a 0.22-micron filter and kept at −20°C for storage. Before being applied, these medicines are diluted with culture media at a ratio of 1:1000. Cell samples are treated with medicines for 24 hours.

### Metabolic activity

The drug concentration and the metabolic activity of cells are calculated by MTT assay after 24 hours of treatment to eliminate the effect of drug. First, a total number of $5\times 10^{3}$ cells of both cell lines (treated and control) are seeded in 96-well plates and incubated overnight. The cell supernatant is removed and after adding the MTT solution in a 37°C incubator for 4 hours, the cells are given sufficient time to metabolize the tetrazolium substance. Then, the supernatant is removed and dimethyl sulfoxide (DMSO) is added to the wells to dissolve the formazan. Finally, the amount of color in each well, which is proportional to the number of cells, is recorded with a spectophotometer at a wavelength of 570nm. GraphPad Prism (version 10.10) is used to generate dose-response curves by performing nonlinear regression analysis [61]. Equation 1 is used to fit the concentration-viability data.


$$\frac{f_{a}}{f_{u}}={\left(\frac{D}{D_{m}}\right)}^{m}\to log\left(\frac{f_{a}}{f_{u}}\right)=mlog\left(\frac{D}{D_{m}}\right)$$ (1)

Here, $f_{a}$ is the fraction of affected cells, $f_{u}(=1-f_{a})$ is the fraction of unaffected cells, D is the dose of drug, $D_{m}$ is the required dose and m denotes the slope of the median-effect plot signifying the trend of the dose-effect curve. m=1, m>1 and m<1 indicate hyperbolic, sigmoidal and planar sigmoidal fittings, respectively.

### Cell viability (live/dead) assay

Cell viability is assessed using the acridine orange assay, in which cells are stained with acridine orange dye. Live cells exhibit green fluorescence under a fluorescence microscope, signifying intact cellular membranes and active metabolism. The live and dead cells are determined using a fluorescence-based kit after 24 hours of treatment. Briefly, orange solution (Sigma 9231A) is placed on cells for 15 minutes at room temperature. Then, they are washed 3 times with PBS (4417P-Sigma). Next, PI (4864P-Sigma) is added to the cells and the cells are washed 3 times with PBS. After 30 minutes, live and dead cells are imaged using a fluorescent microscope (Olympus IX70). The cell viability percent is calculated by counting green points representing live cells and dividing that by the total number of cells.

### Young’s modulus of cells

The atomic force microscopy (AFM, JPK Nanowizard 3) is used to measure the elastic modulus of cells. Initially, 6-well plates containing $5\times 10^{3}$ cells are seeded with cover glass and left overnight to culture. Following PBS washing, test samples are treated with medication solutions mixed into the culture medium, cultured for 24 hours, aspirated, and then they are replaced with the new medium. The cells are loaded using the AFM and force versus deflection is recorded and used to calculate the Young’s modulus of treated and control cell groups. Every sample is examined in 5–8 cells, and a force–displacement curve with 90–175 datapoints is recorded. At the nucleus and neighboring locations, an indentation is made on a 1 µm-square section of the cell sample while the cantilever velocity is set at 2 µm/s. The Hertz model is fitted to the force-displacement curve and Young’s modulus is calculated using the modified Hertz constitutive equation [48].


$$F=\frac{4\sqrt{R}E}{3\left(1-\nu^{2}\right)}\delta^{\frac{3}{2}}$$ (2)

Here, F describes the applied force, ν indicates Poisson’s ratio which is assumed to be 0.5 due to incompressibility, R and δ are cantilever effective radius and indentation depth, respectively. Fig S1 in S1 File illustrates the operational principle of the AFM apparatus and a typical force–deflection curve as an example of the AFM output.

### Nucleus staining and fluorescent microscopy

After seeding 5 × 10³ cells per well on cover glass in 6-well plates, the prescribed medication concentration is added to each well, and the plates are incubated for 24 hours. Cells are fixed for ten minutes after incubation using a 3.8% paraformaldehyde solution. After fixation, excess paraformaldehyde is removed by suction, and two 5-minute PBS-solution washes are carried out. Triton X-100 and 1% BSA are used to permeabilize frozen cell membranes and decrease non-specific hydrophobic binding following the removal of the PBS solution. Coverslips are treated for an hour. This is followed by a two-step PBS-solution wash. All samples are exposed to DAPI for 30 minutes, and then, they are washed twice with PBS solution to stain the nuclei. To examine the cytoskeleton structure of cells, F-Actin, focal contacts and nucleus counterstaining are examined with TRITC-conjugated phalloidin, anti-vinculin and DAPI respectively (FAK 100 package is purchased from Merck). Cell lines are treated with drugs and their cytoskeleton structures are examined by staining with TRITC-conjugated phalloidin, anti-vinculin and DAPI. Using an Olympus IX70 fluorescent microscope, F-actin arrangement and content, and nucleus size, are obtained.

### Quantitative analysis of cell structure and geometry

Nucleus area, F-actin content and arrangement before and after medication are evaluated using FIJI-ImageJ program. For actin filaments distribution, ten segments are carefully selected to divide a section perpendicular to the principal axis on the enclosing ellipse of each cell. By figuring out the average intensity for every section, actin distribution and intensity are quantified.

### Statistical analysis

Lilliefors significance correction is used in conjunction with the Kolmogorov-Smirnov test for pre-statistical analysis data distribution verification. The power transformation approach is utilized to maintain the mean value and standard deviation, while normalizing non-normally distributed data. Significant differences between paired groups are identified using a T-test, and one-way and two-way ANOVA are used to assess differences between the test groups.

## Results and discussion

The way that PCa interacts with the ECM is crucial to explore the cancer behavior and progression. Research has indicated that the presence of ECM components in the tumor microenvironment has a substantial impact on PCa invasion, proliferation, and treatment resistance [55]. The ability of decellularized ECM to function as an antigenic depot and foster protective immunity against tumor recurrence and metastasis underscores the potential benefits of ECM-based strategies in cancer immunotherapy [56]. Moreover, using prostate organoids from patients on different ECM substrates has provided critical new understandings of disease pathology, highlighting the significance of the ECM in PCa behavior [57], including drug resistance, metastasis, and proliferation [32,55,58–61]. Therefore, elucidating the mechanisms underlying PCa progression and developing targeted therapeutic approaches require a thorough understanding of the complex interactions between PCa cells and the elastic modulus of the ECM.

This section describes the prostate cancer cells behavior with different metastatic potential: low (LNCaP), medium (DU145), and high (PC3). The cells are cultured on three substrates with different elastic moduli (stiff, medium, and soft) and treated with three drugs including Docetaxel, Bicalutamide, and Abiraterone Acetate. The MTT assay is performed to calculate drug concentrations needed for treating cancer cells and the live/dead assay is performed to evaluate the viability of the treated cells. Additionally, the actin filament content and arrangement, along with the elastic modulus of cells, are analyzed to assess the impact of treatment on cytoskeletal structure and nucleus size.

### Treatment assessment by MTT assay

**Fig 4:**
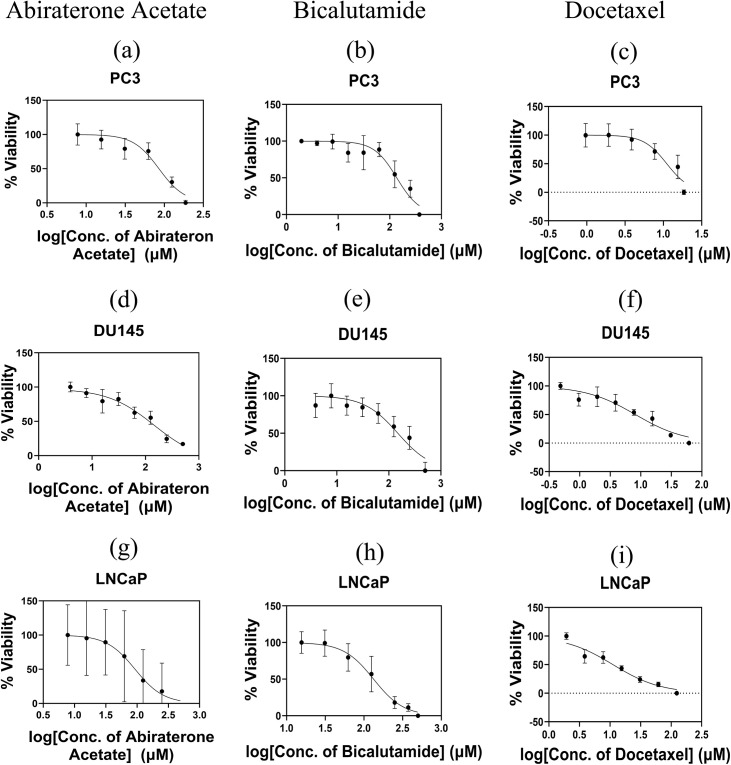
Dose-response curves and the IC50 values. **PC3, DU145 and LNCaP cells are exposed to either (a), (d) and (g) Abiraterone Acetate,** (b), (e) and (h) Bicalutamide or (c), (f) and (i) **Docetaxel for 24 hours, followed by the MTT assay to assess the cell viability.** To calculate the concentration of IC50, the best fitting is performed using the Chou method [49,50].

The IC50 concentration is assessed using the MTT assay. The viability of treated cells is normalized to that of untreated control groups, and the resulting cell viability fractions are plotted against drug concentration on a logarithmic scale (Fig 4). Figs 4a–4c illustrate the cell viability fractions of the PC3 cell line following treatment with Abiraterone Acetate, Bicalutamide, and Docetaxel, respectively. Similarly, Figs 4d–4f depict the cell viability fractions of the DU145 cell line treated with the same drugs. Additionally, Figs 4g–4i present the MTT assay results for the LNCaP cell line following treatment with Abiraterone Acetate, Bicalutamide, and Docetaxel, respectively. The unified theory [49,50] is used to evaluate the IC50 of Docetaxel, Abiraterone Acetate and Bicalutamide drugs in PC3, DU145 and LNCaP cells lines. The mean IC50 values for different treated cell lines are reported in Table 2.

**Table 2:** The IC50 values of docetaxel, abiraterone acetate and bicalutamide in PC3, DU145 and LNCaP cell lines.

	PC3	DU145	LNCaP
	IC50 (µM)		
Abiraterone Acetate	85.34	144.6	93.03
Bicalutamide	137	149.3	133.5
Docetaxel	11.09	8.16	11.21

### Cell viability

**Fig 5:**
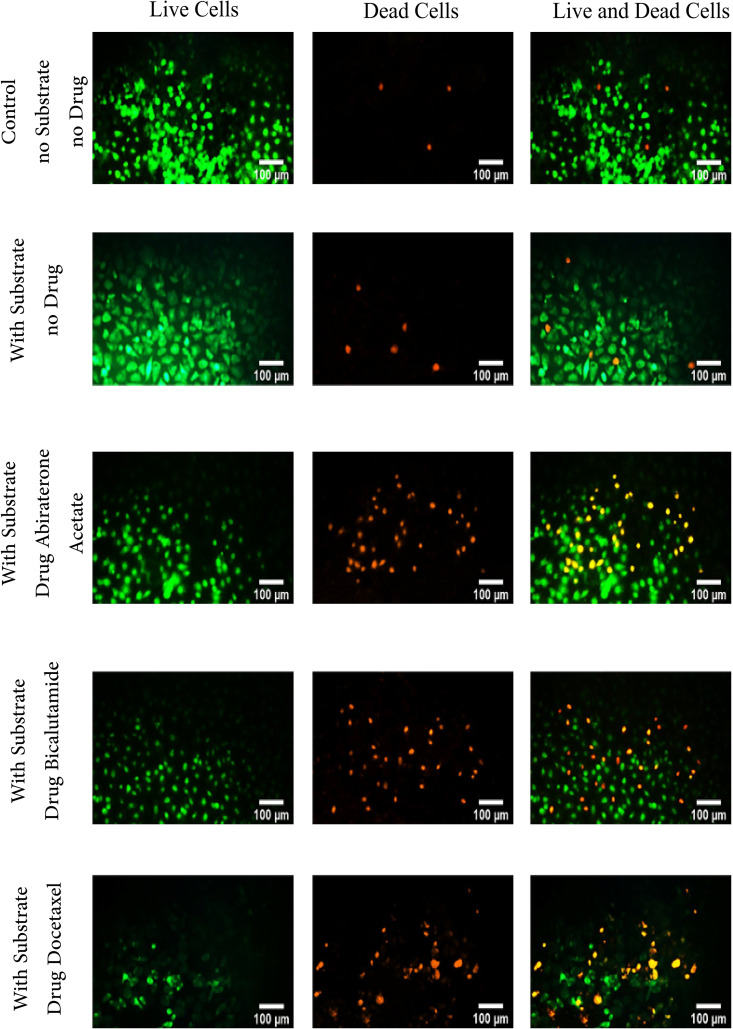
Live/dead images of prostate cancer cells with different metastatic potential (PC3, DU145 and LNCaP) on soft substrate before and after treatment with three drugs (Docetaxel, Abiraterone Acetate, and Bicalutamide); Green indicates live cells, and red indicates dead cells. Each experiment is performed in triplicate (n = 3) for each group.

**Fig 6:**
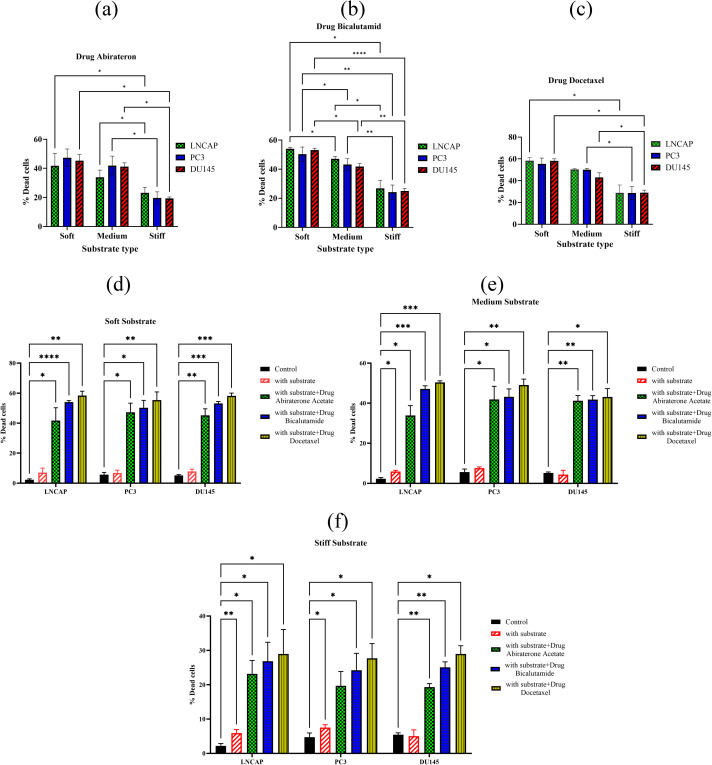
The percentage of dead cells evaluated in live/dead assay on substrates of varying elastic modulus and for three drugs, (a) Abiraterone Acetate, (b) Bicalutamide and (c) Docetaxel and the percentage of dead cells evaluated in the live/dead assay in control group and on substrates of varying elastic modulus, (a) soft, (b) medium and (c) stiff before and after medication. The stars indicate statistical difference between different groups and the control group. (* P < 0.05, ** P < 0.01, *** P < 0.001, **** P < 0.0001).

In the live/dead assay, the viability of three cancer cell lines (PC3, DU145, and LNCaP) is evaluated across three substrate stiffness conditions (soft, medium, and stiff) and following treatment with three drugs (Docetaxel, Abiraterone Acetate, and Bicalutamide). As an example, live/dead images of DU145 cells on a soft substrate are shown in Fig 5. Using ImageJ software, the images are analyzed to assess differences in color contrast, allowing for the calculation of cell viability. Figs 6a–6c show the rate of cell death on different substrates following treatment with Abiraterone Acetate, Bicalutamide and Docetaxel, respectively. Apparently, the rates of cell death on the stiff substrates are lower than the corresponding values on the soft substrates. Subsequently, Figs 6d–6f illustrate the effect of substrate elastic modulus on cell viability for soft, medium, and stiff substrates, respectively. According to the results, a significant difference (P<0.05) can be seen in the percentage of dead cells among cells cultured on stiff substrates (20%−30%) compared to soft substrates (40%−60%). This trend is seen among all cell lines, and for all types of medications. Additionally, based on the statistical analysis, there is a significant difference between all groups with the control group (untreated cells and without substrate). On soft substrates, across all cell lines (PC3, DU145, and LNCaP), there is no significant difference (P > 0.05) between treated and untreated cells cultured on the substrate and also between untreated cells on the substrate and the control group (untreated cells without substrate). However, a significant difference is observed between the control group and all other groups. Furthermore, when medium substrates are used, for PC3 and DU145 cell lines, there is no significant difference (P>0.05) between the group of untreated cells on the substrate and the control group. Nevertheless, a significant difference is observed between the control group and all other groups.

On the stiff substrates, for DU145 cell line, there is no significant difference (P>0.05) between the untreated cells on substrate and the control group. Also, for PC3 cell line, insignificant difference exists between the cells on the substrate and treated by Abiraterone Acetate and the control group. However, there is significant difference between the control group and all other groups. In addition, among drugs, the effect of Docetaxel drug (targeting the cytoskeleton structure of cells) is more significant than the other two drugs (Abiraterone Acetate and Bicalutamide), which is observed for all the three cell lines.

The inconsistency between live/dead and IC50 results on soft substrates can be related to incorrect consideration of a part of apoptotic cells as live cells in the MTT test and also experimental errors. Such inconsistency is more significant for LNCaP cell line treated with Abiraterone Acetate drug, having more apoptotic cells.

The obtained results are consistent with prior studies showing that increased elastic modulus of the ECM decreases the treatment efficacy and apoptosis triggered by chemotherapy. The enhanced resistance linked to stromal elastic modulus interferes with the delivery of chemotherapeutic drugs [62,63]. Additionally, research has shown a direct relationship between the elastic modulus of substrate and the antiproliferative properties of chemotherapy medications [64]. Based on the findings, cancer cells proliferate more rapidly when the substrate is stiffer.

**Fig 7:**
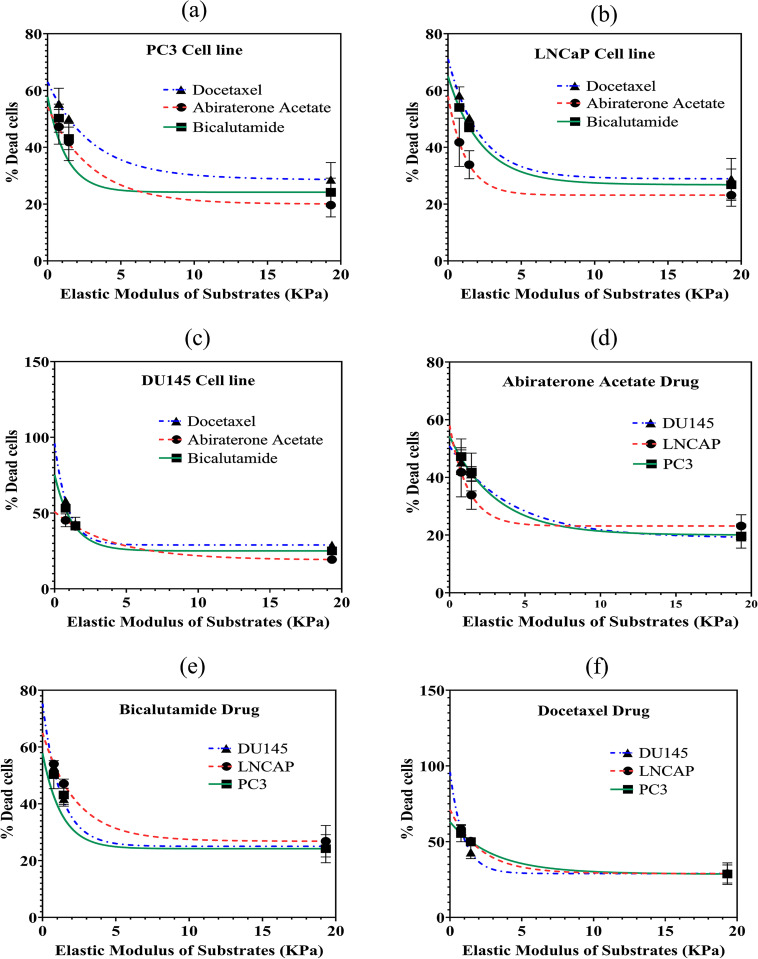
The percentage of dead cells versus the elastic modulus of substrates for: (a) to (c) three cell lines of PC3, LNCaP and DU145, respectively; (d) to (f) three drugs of Abiraterone Acetate, Bicalutamide and Docetaxel, respectively.

In Fig 7, the percentage of dead cells is plotted versus the elastic modulus of the substrates. Figs 7a–7c display the percentage of dead cells as a function of substrate elastic modulus for the PC3, LNCaP, and DU145 cell lines, respectively. Additionally, Figs 7d–7f present the percentage of dead cells versus substrate elastic modulus for treatments with Abiraterone Acetate, Bicalutamide, and Docetaxel, respectively. As seen, for all cell lines and treatments, the lowest cell death belongs to hard substrate. Also, for stiff substrate, Docetaxel has the most impact on all three cell lines (PC3, LNCaP and DU145), with quite similar impacts on different cells. The drug impact shows variations for the three cell lines on soft substrate.

### Quantitative analysis of structural features

This section focuses on structural features, including cytoskeletal structure and the nucleus size, to assess the impact of medication in relation to mechanical properties.

### Actin filament content

**Fig 8:**
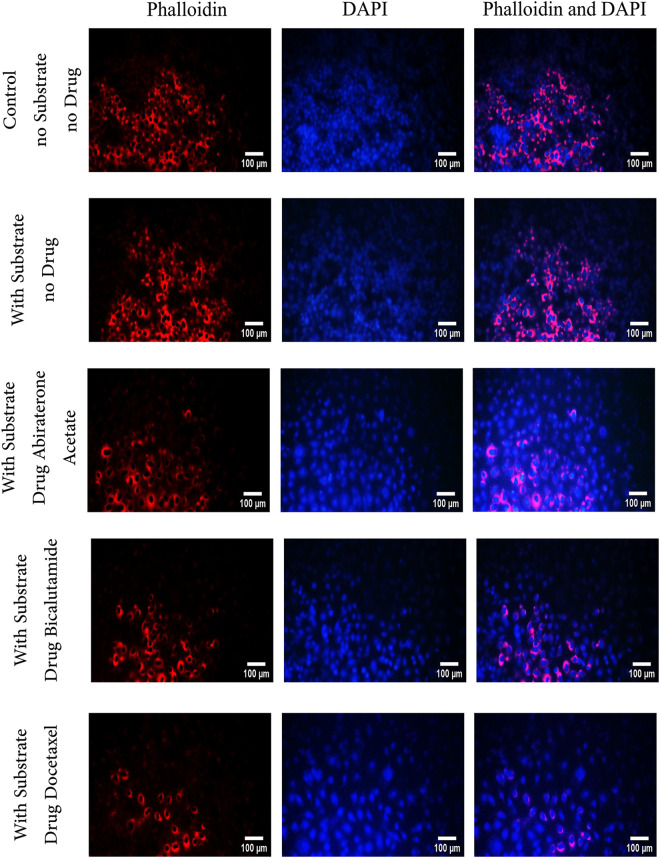
Staining Phalloidin/DAPI of DU145 cells on a stiff substrate. The red color indicates the cytoskeletal content and the cytoskeleton structure of the cells, and the blue color indicates the nucleus of cells. Each experiment is performed in triplicate (n = 3) for each group.

**Fig 9:**
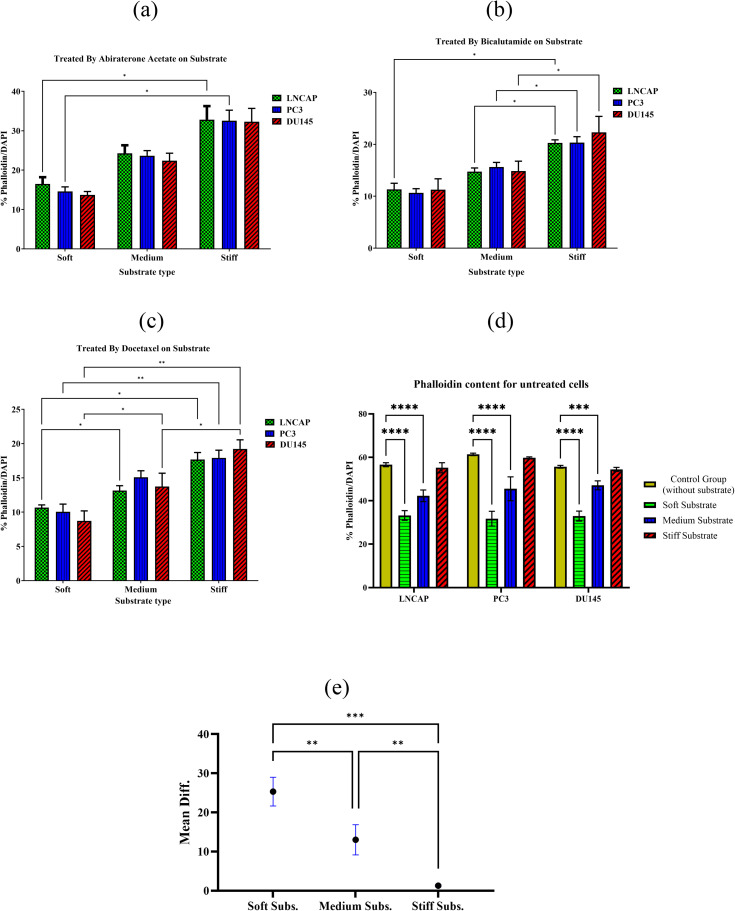
The percentage of Phalloidin/DAPI on different substrates using three drugs including (a) Abiraterone Acetate, (b) Bicalutamide and (c) Docetaxel. (d) Phalloidin content in untreated cells: comparison between the control group and cells cultured on hydrogel substrates (* P < 0.05, ** P < 0.01, *** P < 0.001, **** P < 0.0001), (e) mean difference in phalloidin content between the control group and untreated cells cultured on the test substrates.

Three cancer cell lines (PC3, DU145 and LNCaP) cultured on three substrates (soft, medium and stiff) are treated with three drugs (Docetaxl, Abiraterone Acetate, and Bicalutamide) and the cytoskeleton structure and the cell nucleus are stained with phalloidin/DAPI. Fig 8 presents phalloidin/DAPI-stained images of DU145 cells on a stiff substrate, serving as a representative example across all groups (various cell lines and substrate conditions). The images are analyzed using ImageJ software, and the percentage of cytoskeletal actin content is quantified based on differences in color contrast. Figs 9a–9c show the cytoskeletal actin content on different substrates following treatment by the three drugs of Abiraterone Acetate, Bicalutamide and Docetaxel, respectively. As observed, the phalloidin content in treated cell lines cultured on a soft substrate ranges from 8% to 16%, whereas on a stiff substrate, it ranges from 17.6% to 32%. This indicates that cells grown on a stiff substrate exhibit higher phalloidin levels, contributing to increased resistance to metastasis.

Meanwhile, as shown in Fig 9d, the group of untreated cells cultured on stiff substrates across all cell lines does not exhibit a significant difference (P > 0.05) compared to the control group (untreated cells without a substrate). However, all other groups of untreated cells cultured on soft and medium substrates show a significant difference from the control group. Additionally, among the untreated cells, phalloidin content is higher in the control groups than in those cultured on hydrogel substrates. However, this difference diminishes for stiff substrates, as shown in Fig 9e.

Findings of the current study are consistent with those reported in previous experimental studies. Notably, the cytoskeletal architectures of SKOV-3 cells grown on different substrates were investigated using confocal laser scanning microscopy [65]. The results indicated that stress fibers are distinctly visible in cells cultured on more rigid substrates, whereas F-actin and tubulin fluorescence appear faint in cells on softer substrates. It was reported that variations in substrate elastic modulus influence the production and structural organization of several cytoskeletal proteins, including vimentin, microtubulin, and F-actin. Another study on wound healing demonstrated that cells cultured on a 35kPa substrate exhibit greater migratory potential compared to those on a 3kPa substrate. It was reported that actin depolymerization reduces cellular flexibility while increasing viscosity, ultimately impairing migration capacity [32]. Matrix elastic modulus and biological forces are key determinants of cell morphology, representing the primary internal and external biological forces influencing cellular behavior. Rigid substrates offer enhanced physical support for cell growth, promoting increased cell adhesion and cytoskeletal development [66]. A positive correlation between matrix elastic modulus and cell spreading area has been observed in various normal human and cancer cell types cultured in vitro [67–69].

### Actin filament arrangement

To investigate the effect of drug on actin filament arrangement, two cell lines of PC30 (invasive) and LNCaP (non-invasive) cell lines are cultured on the stiff substrate and subjected to Docetaxel treatment. The actin filaments and the cytoskeletal structure of the cells are stained, photographed, and analyzed before and after the treatment.

**Fig 10:**
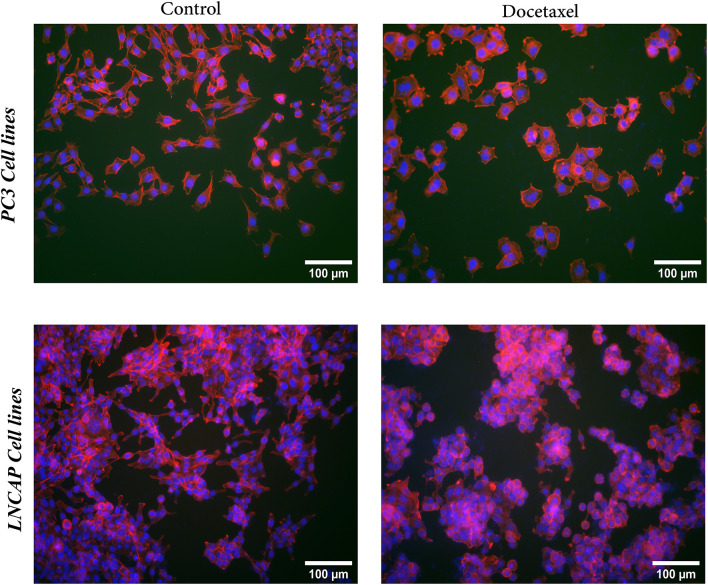
Comparison of actin filament distribution in cells with low (LNCaP) and high (PC3) metastatic potential before and after drug treatment; the cells are cultured on stiff substrate and treated with Docetaxel drug.

As shown in Fig 10, untreated PC30 cell lines exhibit extensive cytoskeletal organization around the nucleus, with the filamentous structure radiating in all directions. After the treatment, the density of the cytoskeletal content decreases which results in the shrinkage of the cytoskeleton structure. Our experimental findings indicate that, prior to treatment, most LNCaP cells exhibit a stretched cytoskeletal structure oriented along two distinct directions. After the treatment, due to the shrinkage of the cytoskeleton structure, the cells tend to stick together and form a colony.

**Fig 11:**
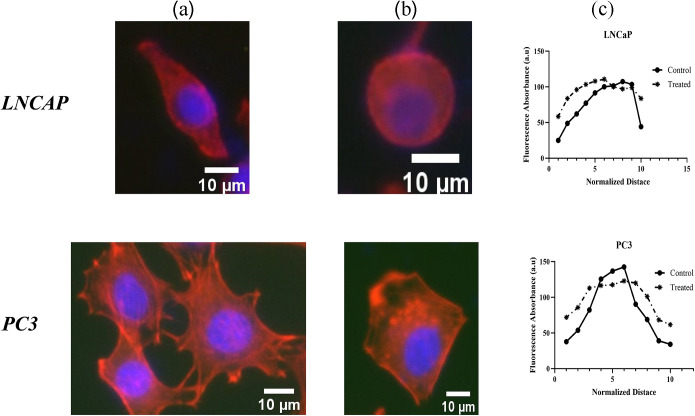
Actin filaments distribution in PC3 and LNCaP cells cultured on stiff substrates. **(a)** Control group (untreated), **(b)** treated with Docetaxel, **(c)** average actin filament distribution along a direction perpendicular to the major (long) axis, passing through the center of a representative PC3 and LNCaP cell before and after treatment.

Fig 11 illustrates the distribution of actin filaments in PC3 and LNCaP cell lines cultured on the stiff substrate. Fig 11a illustrates the control group (untreated cells), while Fig 11b presents cells treated with Docetaxel. Additionally, Fig 11c depicts the average distribution of actin filaments along a line perpendicular to the major (long) axis, passing through the center of a cell lines before and after treatment. The amount of cytoskeletal content is quantified using a method based on the fluorescent intensity along lines perpendicular to the main axis of the cell, as presented by Zonderland et al. [70]. The results indicate that the medications induce significant alterations in actin protein organization, affecting both the cytoplasmic domain and cell boundaries. As visualized in Figs 11a–11c, the Docetaxel drug which targets the cytoskeleton structure, exhibits a weaker effect on the cytoskeleton of the LNCaP cell line compared to the more metastatic PC30 cell line.

Previous studies support the findings of the present study, demonstrating that substrate stiffness significantly influences cytoskeletal organization and migratory behavior in cancer cells. Prostate cancer cell lines, such as DU145 and PC3, exhibit distinct morphological adaptations when cultured on substrates of varying stiffness (e.g., 0.75 MPa and 2.92 MPa), leading to altered growth patterns [71]. Moreover, increased substrate stiffness (e.g., 35 kPa) enhances the aggregation of F-actin filaments and reinforces cell polarity—an essential factor in facilitating directional migration [32]. Consistently, cells cultured on stiffer substrates exhibit elevated migration rates, as demonstrated by wound healing assays, underscoring a direct correlation between substrate stiffness and metastatic potential [32,73].

Additionally, substrate rigidity modulates the expression of neuropilin-1 (NRP1), a key molecule implicated in cell migration and malignancy, thereby influencing the migratory capacity of prostate cancer cells [73]. Notably, the mechanical properties of the substrate also impact cellular responses to drug treatments. Specifically, stiffer environments not only exacerbate the invasive behavior of cancer cells but also diminish the efficacy of therapeutic interventions [74].

### Size of nucleus

To examine the impact of chemically distinct drugs on nucleus size in cells with high and low metastatic potential, the nucleus size of PC30 and LNCaP cells cultured on stiff substrates is evaluated before and after treatment with Docetaxel and Bicalutamide. Measurements indicate that the nucleus size of PC30 and LNCaP cells is 154.49 μm² and 95.839 μm², respectively, demonstrating that the more invasive PC30 cells (high metastasis potential) possess a larger nuclear size compared to the less invasive LNCaP cells (low metastasis potential). Following drug treatment, invasive cell lines exhibit a more pronounced reduction in nucleus size compared to non-invasive ones. According to the results, Docetaxel treatment reduces nucleus size by 15.99% in PC30 cells and 14.92% in LNCaP cells. Bicalutamide induces further reductions of 43.01% and 32.91% in PC30 and LNCaP cells, respectively, highlighting its stronger impact on nuclear size reduction compared to Docetaxel.

**Fig 12:**
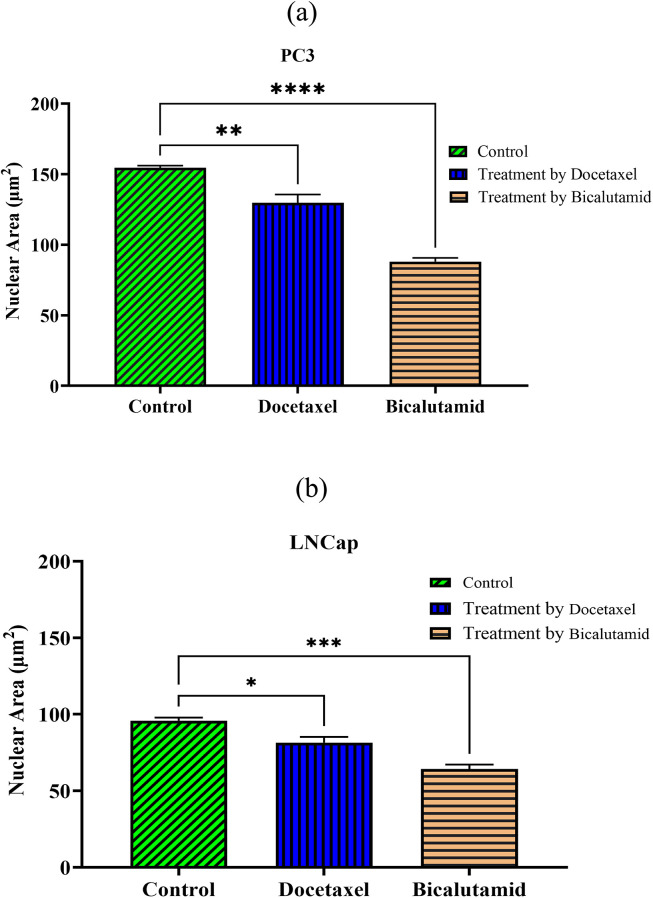
Nucleus size of (a) PC3 cells and (b) LNCaP cells. Each experiment is conducted with 140 samples per group (n = 140). Data are presented as mean ± standard deviation (SD). Normality is assessed using the Shapiro-Wilk test, and statistical analysis is performed using one-way ANOVA to evaluate differences among groups. The control group is compared with the experimental groups, and asterisks indicate statistically significant differences (P < 0.05, ** P < 0.01, *** P < 0.001, **** P < 0.0001).

Fig 12 presents nucleus area measurements in PC3 and LNCaP cell lines before and after treatment with Bicalutamide and Docetaxel. Fig 12a illustrates nucleus area for PC3 cells, while Fig 12b depicts the nucleus area of LNCaP cells under identical treatment conditions. Each group consists of approximately 140 analyzed samples. As seen, the nucleus size of the treated cell lines decreases compared to the control group for both cell lines. A significant difference is observed between the treated and control groups for both cell lines. Table 3 presents the mean nucleus size and the corresponding rate of size reduction across different conditions.

**Table 3:** Mean nucleus size and corresponding rate of size reduction across experimental conditions.

		Mean of Nucleus Area [$\mu m^{2}$]		
		Control	Docetaxel	Bicalutamide
PC3	Mean of Nucleus Area [$\mu m^{2}$]	154.49±4.17	129.778±11.75	88.045±5.12
	Reduction rate %		15.996%	43.009%
LNCaP	Mean of Nucleus Area [$\mu m^{2}$]	95.839±3.55	81.544±7.39	64.298±5.64
	Reduction rate %		14.92%	32.91%

### Elastic modulus of cells

**Fig 13:**
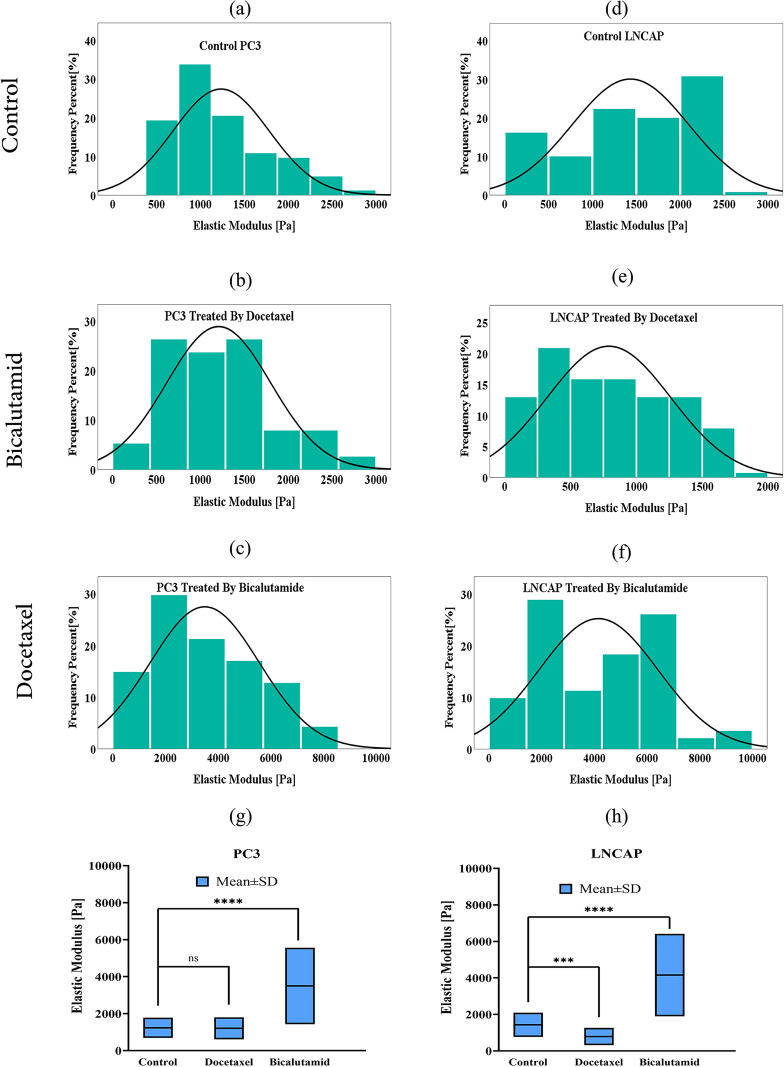
The elastic modulus from 141 measurements for PC3 (left column) and LNCaP (right column) cell lines cultured on stiff substrates using AFM: (a), (d) untreated cells, (b), (e) treated with Docetaxel and (c), (f) treated with Bicalutamide. Box-and-whisker plot illustrating the elastic modulus of **(g)** PC3 and **(h)** LNCaP cell lines cultured on stiff substrates, before and after treatment. *** P < 0.001, **** P < 0.0001.

To investigate the impact of drug treatment, the elastic modulus of two prostate cancer cell lines—one invasive (PC3) and one non-invasive (LNCaP)—is measured before and after treatment. The cells are cultured on stiff substrates and exposed to two therapeutic agents, Docetaxel and Bicalutamide, to assess changes in mechanical properties in response to treatment. The elastic modulus of cells is determined using AFM measurements taken at 141 points for each cell group. The results are presented in Figs 13a–13f, illustrating variations under different experimental conditions: untreated cells (a, d), cells treated with Docetaxel (b, e), and cells treated with Bicalutamide (c, f). Additionally, the mean elastic modulus, calculated based on these measurements, is depicted in Figs 13g and 13h, corresponding to PC3 and LNCaP cells before and after treatment, respectively. As observed, the mean elastic modulus for LNCaP cells is 1234.91 Pa, which is lower than that of PC3 cells at 1433.45 Pa. Additionally, treatment with Docetaxel results in a decrease in the elastic modulus for both cell lines compared to the control group, though the reduction in PC3 cells is relatively minor. These findings suggest that the cytoskeletal disruption induced by Docetaxel may contribute to the observed changes in cellular mechanical properties. On the contrary, the elastic modulus of the cells increases for both cell lines after the treatment with Bicalutamide compared to the control group. Such an increase is significant for both cell lines and it can be attributed to the release of the androgen receptor proteins. Table 4 displays the variations in the elastic modulus of cells following medication. Based on the results, changes in elastic modulus of LNCaP cells after the treatment more pronounced in comparison to PC3 cells. These findings are consistent with existing literature [75–77]. Notably, the Young’s modulus of HPV-PZ-7 and PC3 cells has been observed to decrease following treatment with Blebbistatin when cultured on stiff substrates, whereas no significant effect has been reported on soft substrates. This outcome suggests that inhibition of actin polymerization by an actin-binding protein inhibitor prevents the formation of actin fibril bundles. As a result, prostate cancer cells exhibit reduced elasticity and increased viscosity, ultimately leading to diminished migratory capacity [32,72].

**Table 4:** Mean elastic modulus of cells cultured on stiff substrates, along with the percentage increase or decrease observed before and after treatment with Docetaxel and Bicalutamide.

Cell line	Parameter	Control	Docetaxel	Bicalutamide
PC3	Mean of Young’s Modulus [Pa]	1433.4549	1208.7221	3496.7272
	Decrease/increase percentage		−15.68%	143.94%
LNCaP	Mean of Young’s Modulus [Pa]	1234.91	791.999	4157.6105
	Decrease/increase percentage		−35.86%	236.67%

Previous studies emphasized the significant influence of substrate stiffness on both cancer and normal cell behavior. In neural cells, soft substrates were shown to improve cell viability and enhance neuroprotective drug responsiveness through EGFR/PI3K/AKT signaling pathways. Additionally, increased substrate stiffness was linked to heightened proliferation and chemotherapy resistance, accompanied by alterations in gene expression and mechanical properties. Furthermore, stiffer substrates were observed to facilitate migration and angiogenesis in lung cancer models, while physiological stiffness helps maintain the phenotype of keratocytes, underscoring the crucial role of matrix properties in tissue engineering [64,73–75].

However, while these studies individually explored either proliferation, drug resistance, or migration in relation to substrate stiffness, the present study provides an integrated analysis by simultaneously evaluating drug efficacy (IC50), cytoskeletal rearrangements, nucleus morphology, and mechanical properties (Young’s modulus) across prostate cancer cells with different metastatic potentials. Additionally, by combining multiple anticancer drugs with distinct cellular targets and analyzing their effects across soft, medium, and stiff substrates, new insights are gained. This comprehensive approach provides a broader understanding of how mechanical cues and chemical treatments synergistically modulate cancer cell behavior, offering new insights for designing optimized therapeutic strategies.

The findings suggest that distinct molecular mechanisms govern the effects of anticancer drugs and substrate elasticity on prostate cancer cells. Cellular responses to substrate stiffness are regulated through mechanotransduction, highlighting the influence of mechanical cues on cancer cell behavior. As integrins interact with extracellular matrix proteins, they activate kinases, which in turn trigger signaling through the PI3K/AKT and RhoA/ROCK pathways. These pathways control cytoskeletal dynamics, cell survival, and proliferation [76,77].

Key regulators play a crucial role in controlling actin cytoskeleton dynamics and mediating cellular responses to mechanical stimuli and drug treatments. These factors influence cytoskeletal reorganization, impacting various aspects of cancer cell behavior, including migration, adhesion, and mechanotransduction. Rac1 and Cdc42 control the production of lamellipodia and filopodia, which facilites cell migration and invasion, while RhoA/ROCK signaling enhances actin stress fiber formation and contractility, which is more noticeable on rigid substrates [78,79]. Actin polymerization and depolymerization are regulated by proteins which affect the cell motility and shape. Variations in the F-actin content point to modifications in these proteins’ activities [80,81].

Changes in nucleus area and mechanics suggest the involvement of regulatory processes governing nucleus structure and function. The nucleus envelope and cytoskeleton are connected by the Linker of the Nucleoskeleton and Cytoskeleton (LINC) complex, which transmits mechanical signals that influence chromatin architecture and gene expression [82,83]. Variations in nucleus mechanics can impact chromatin accessibility and histone alterations, which in turn can impact transcriptional control [84,85]. These molecular pathways can explain our findings about diverse pharmacological effects on mechanical characteristics, cytoskeletal structure, and cell death. For example, stronger mechanotransduction signaling may lead to greater breakdown of cytoskeletal integrity and higher rates of apoptosis, which could explain why Docetaxel is more effective on softer substrates. Likewise, the more pronounced effect of Bicalutamide on the reduction of nucleus area implies a noteworthy regulation of androgen receptor signaling and related nucleus processes.

The interaction between the YAP/TAZ signaling pathway and integrin-mediated pathways plays a key role in the response of cancer cells to changes in the ECM elasticity. As ECM elasticity increases, mechanotransduction mechanisms are activated which promote proliferation, migration, tumor growth, and drug resistance. This response is mainly regulated by the Hippo pathway, in which YAP and TAZ act as central transcription factors and translocate to the nucleus to increase the expression of genes associated with survival and metastasis [86,87]. In colorectal cancer-associated fibroblasts, dysregulation of the Hippo pathway as a result of high ECM stiffness has been shown to increase YAP/TAZ activity and accelerate tumor growth [88]. In addition, integrins play an important role in mechanical signal transduction by directly connecting the ECM to the cytoskeleton and facilitating YAP/TAZ activation through pathways such as FAK and Rho [87,89]. Increased integrin expression in tumor fibroblasts also promotes cell invasiveness and accelerates malignant progression [87,89]. Targeting YAP/TAZ and integrin signaling has been proposed as a promising therapeutic approach to reduce the negative effects of ECM stiffness on cancer progression and drug resistance [87]. YAP/TAZ inhibitors have been shown to reduce metastasis potential in preclinical models, highlighting the importance of the role of mechanical transduction in cancer therapy. However, it should be noted that due to the high complexity of the tumor microenvironment and the existence of compensatory mechanisms, the complete success of these treatments may face challenges.

Recent studies have also shown that prostate cancer cells show a significant increase in YAP/TAZ nuclear translocation in response to increased substrate stiffness, which leads to increased cell migration and proliferation, especially in bone metastasis-derived cells [90]. Meanwhile, the β1-integrin–ILK–CDC42–N-WASP signaling pathway is critical for TAZ nuclear translocation and induces the expression of stemness genes such as NANOG and OCT4, which play a key role in the initiation of metastatic processes. Furthermore, TAZ is sensitive to mechanical stresses such as fluid shear stress, and its activation under these conditions increases cell proliferation and motility, highlighting the role of TAZ in the adaptation of cancer cells to the mechanical forces of the tumor microenvironment [91].

Further evidence suggests that prostate cancer cells have heterogeneous responses to mechanical inputs, such that metastatic target tissues (bone, lymph, etc.) exhibit different patterns of migration and proliferation depending on the stiffness of the substrate [90]. For example, in softer environments, lymphatic metastasis-derived cells tend to cluster more and increase expression of the CD44 marker, whereas in stiffer substrates, this behavior is reduced and the migration pattern changes [90].

### Limitations

Despite the insights provided by the present study on the influence of substrate mechanical properties on prostate cancer cell responses to chemotherapeutic drugs, several limitations should be considered. First, the study was conducted exclusively in vitro, without in vivo evaluations. This may restrict the generalizability of the findings, as the in vivo environment incorporates complex factors such as immune interactions, blood flow dynamics, cellular crosstalk, and hormonal signaling, all of which can significantly impact cellular responses to drugs and the extracellular matrix.

Second, synthetic hydrogels were employed and fibronectin coating was used to ensure consistent cell adhesion and enhance physiological relevance. While these substrates allow precise mechanical control, they do not fully replicate the biological conditions of native tissues.

Third, drug penetration into polyacrylamide matrices was not experimentally assessed, and variations in permeability may influence drug efficacy outcomes. Additionally, the use of only three prostate cancer cell lines with differing metastatic potential and three specific drugs may limit the broader applicability of the findings.

## Conclusions

The present study clarified the complex interactions between anticancer medications and elastic modulus of substrate on prostate cancer cells of various metastatic potentials. We utilized PAAM substrates with three distinct elastic moduli to replicate and imitate the range of elastic modulus detected by cells. Also, Docetaxel, Bicalutamide and Abiraterone Acetate drugs were selected as medications across all substrate elasticity levels for PCa treatment. The impact of the study parameters including the substrate elastic modulus, the chemical drugs and the degree of cells invasion were investigated on the cytoskeleton structure of cells, the size of nucleus, and the viability of cells.

The notable result on the impact of substrate revealed higher rate of cell death cultured on hard substrate compared to soft and medium ones. Additionally, the administration of medications caused noteworthy changes in the nucleus morphology and cytoskeletal architecture, as demonstrated by a considerable post-treatment shrinkage. In this regard, the noteworthy impact of the anticancer medications revealed the effective role of Docetaxel and Bicalutamide in decreasing the nucleus size. The results were more conspicuous using Bicalutamide, indicating different modes of action influencing the cellular activity. Associated with the cytoskeletal changes, the cells’ elastic modulus showed changes that were correlated with their propensity to spread and the effectiveness of their medication, highlighting the critical role of mechanical characteristics in the cancer cells behavior and response to treatments.

The obtained results emphasized the importance of the mechanical properties of cell environment in selecting cancer treatment plans. Based on the findings, the treatment success is significantly impacted by the synergy between the degree of cancer cell invasiveness, the medication and the host tissue elastic modulus. A thorough understanding of such interactions leads to successful therapeutic approaches and improved outcomes. Also, as a practical suggestion in this research, it can be mentioned that for greater effectiveness of drugs, drugs should be used that both affect the ECM of cells and make them softer and also affect the cytoskeleton of cells. Because in the present study, it was observed that the rate of cell death was higher on soft substrates compared to stiff ones. This finding suggests that enhancing drug efficacy could be achieved by targeting the extracellular matrix to reduce its elastic modulus, as drugs demonstrated greater effectiveness on substrates with a low elastic modulus. Additionally, cells treated with Docetaxel, a cytoskeleton-targeting drug, exhibited a higher rate of cell death compared to the other two drugs with different targets. So, as a practical suggestion derived from this study, it is recommended to use drugs that simultaneously target the ECM to reduce its elastic modulus and disrupt the cytoskeleton of cells to enhance their overall effectiveness.

## Supporting information

S1 File Fig S1. (a) Tensile testing on the substrates; the samples are tested in a cylindrical mold with diameter and height of 1 inch under 20 mm per minute strain and 10 N load. Three samples are tested for each elastic modulus, (b) schematic of atomic force microscopy-based elastic modulus measurement of cells; A cantilever applies force to the cells, and a detector measures the resulting deflection, (c) the force-deflection curve as an example of the AFM outputs.
(DOCX)

## References

[1] Ferlay J, Soerjomataram I, Dikshit R, Eser S, Mathers C, Rebelo M, et al. Cancer incidence and mortality worldwide: sources, methods and major patterns in GLOBOCAN 2012. Int J Cancer. 2015;136(5):E359-86. doi: 10.1002/ijc.29210 25220842

[2] Benjaminsen E. Multiple sclerosis in Northern Norway, epidemiology and comorbidity. 2022.

[3] Siegel RL, Miller KD, Jemal A. Cancer statistics, 2018. CA: a cancer journal for clinicians. 2018;68(1):7–30. 29313949 10.3322/caac.21442

[4] Kobayashi M, Ishida H, Shindo T, Niwa S-I, Kino M, Kawamura K, et al. Molecular analysis of multifocal prostate cancer by comparative genomic hybridization. Prostate. 2008;68(16):1715–24. doi: 10.1002/pros.20832 18781578

[5] Armenia J, Wankowicz SAM, Liu D, Gao J, Kundra R, Reznik E, et al. The long tail of oncogenic drivers in prostate cancer. Nat Genet. 2018;50(5):645–51. doi: 10.1038/s41588-018-0078-z 29610475 PMC6107367

[6] Robinson D, Van Allen EM, Wu Y-M, Schultz N, Lonigro RJ, Mosquera J-M, et al. Integrative clinical genomics of advanced prostate cancer. Cell. 2015;161(5):1215–28. doi: 10.1016/j.cell.2015.05.001 26000489 PMC4484602

[7] Massie CE, Mills IG, Lynch AG. The importance of DNA methylation in prostate cancer development. The Journal of steroid biochemistry and molecular biology. 2017;166:1–15. 27117390 10.1016/j.jsbmb.2016.04.009

[8] Disanza A, Steffen A, Hertzog M, Frittoli E, Rottner K, Scita G. Actin polymerization machinery: the finish line of signaling networks, the starting point of cellular movement. Cell Mol Life Sci. 2005;62(9):955–70. doi: 10.1007/s00018-004-4472-6 15868099 PMC11924564

[9] Chang C-Y, Lin H-T, Lai M-S, Shieh T-Y, Peng C-C, Shih M-H, et al. Flexible Localized Surface Plasmon Resonance Sensor with Metal-Insulator-Metal Nanodisks on PDMS Substrate. Sci Rep. 2018;8(1):11812. doi: 10.1038/s41598-018-30180-8 30087401 PMC6081403

[10] Gilchrist CL, Darling EM, Chen J, Setton LA. Extracellular matrix ligand and stiffness modulate immature nucleus pulposus cell-cell interactions. PLoS One. 2011;6(11):e27170. 10.1371/journal.pone.0027170 PMC3210142 22087260

[11] Giretti MS, Fu X-D, De Rosa G, Sarotto I, Baldacci C, Garibaldi S, et al. Extra-nuclear signalling of estrogen receptor to breast cancer cytoskeletal remodelling, migration and invasion. PLoS One. 2008;3(5):e2238. doi: 10.1371/journal.pone.0002238 18493596 PMC2375059

[12] Rossy J, Gutjahr MC, Blaser N, Schlicht D, Niggli V. Ezrin/moesin in motile Walker 256 carcinosarcoma cells: signal-dependent relocalization and role in migration. Exp Cell Res. 2007;313(6):1106–20. doi: 10.1016/j.yexcr.2006.12.023 17292355

[13] Louvet-Vallée S. ERM proteins: from cellular architecture to cell signaling. Biol Cell. 2000;92(5):305–16. doi: 10.1016/s0248-4900(00)01078-9 11071040

[14] Seetharaman S, Etienne-Manneville S. Cytoskeletal Crosstalk in Cell Migration. Trends Cell Biol. 2020;30(9):720–35. doi: 10.1016/j.tcb.2020.06.004 32674938

[15] Lekka M, Pabijan J, Orzechowska B. Morphological and mechanical stability of bladder cancer cells in response to substrate rigidity. Biochim Biophys Acta Gen Subj. 2019;1863(6):1006–14. doi: 10.1016/j.bbagen.2019.03.010 30878701

[16] Zemła J, Danilkiewicz J, Orzechowska B, Pabijan J, Seweryn S, Lekka M. Atomic force microscopy as a tool for assessing the cellular elasticity and adhesiveness to identify cancer cells and tissues. Seminars in Cell & Developmental Biology. Elsevier. 2018. 10.1016/j.semcdb.2017.06.029 28694112

[17] Kuznetsova TG, Starodubtseva MN, Yegorenkov NI, Chizhik SA, Zhdanov RI. Atomic force microscopy probing of cell elasticity. Micron. 2007;38(8):824–33. doi: 10.1016/j.micron.2007.06.011 17709250

[18] Li Z, Lee H, Zhu C. Molecular mechanisms of mechanotransduction in integrin-mediated cell-matrix adhesion. Exp Cell Res. 2016;349(1):85–94. doi: 10.1016/j.yexcr.2016.10.001 27720950 PMC5101173

[19] Sanz-Ramos P, Mora G, Ripalda P, Vicente-Pascual M, Izal-Azcárate I. Identification of signalling pathways triggered by changes in the mechanical environment in rat chondrocytes. Osteoarthritis Cartilage. 2012;20(8):931–9. doi: 10.1016/j.joca.2012.04.022 22609478

[20] Ramezani SR, Mojra A, Tafazzoli-Shadpour M. Investigating the effects of substrate stiffness on half-maximal inhibitory concentration of chemical anticancer drugs, cell viability and migration of cell lines. Cellular, Molecular and Biomedical Reports. 2024;:141–7.

[21] Tilghman RW, Cowan CR, Mih JD, Koryakina Y, Gioeli D, Slack-Davis JK, et al. Matrix rigidity regulates cancer cell growth and cellular phenotype. PLoS One. 2010;5(9):e12905. doi: 10.1371/journal.pone.0012905 20886123 PMC2944843

[22] Mielnicka A, Kołodziej T, Dziob D, Lasota S, Sroka J, Rajfur Z. Impact of elastic substrate on the dynamic heterogeneity of WC256 Walker carcinosarcoma cells. Sci Rep. 2023;13(1):15743. doi: 10.1038/s41598-023-35313-2 37735532 PMC10514059

[23] Jeong J-T, Choi M-K, Sim Y, Lim J-T, Kim G-S, Seong M-J, et al. Effect of graphene oxide ratio on the cell adhesion and growth behavior on a graphene oxide-coated silicon substrate. Sci Rep. 2016;6:33835. doi: 10.1038/srep33835 27652886 PMC5031981

[24] Prein C, Beier F. ECM signaling in cartilage development and endochondral ossification. Curr Top Dev Biol. 2019;133:25–47. doi: 10.1016/bs.ctdb.2018.11.003 30902255

[25] Zhuang Y, Huang Y, He Z, Liu T, Yu X, Xin SX. Effect of substrate stiffness on the mechanical properties of cervical cancer cells. Arch Biochem Biophys. 2022;725:109281. doi: 10.1016/j.abb.2022.109281 35537506

[26] Luo T, Tan B, Zhu L, Wang Y, Liao J. A Review on the Design of Hydrogels With Different Stiffness and Their Effects on Tissue Repair. Front Bioeng Biotechnol. 2022;10:817391. doi: 10.3389/fbioe.2022.817391 35145958 PMC8822157

[27] Ashammakhi N, Ahadian S, Xu C, Montazerian H, Ko H, Nasiri R, et al. Bioinks and bioprinting technologies to make heterogeneous and biomimetic tissue constructs. Mater Today Bio. 2019;1:100008. doi: 10.1016/j.mtbio.2019.100008 32159140 PMC7061634

[28] Trappmann B, Chen CS. How cells sense extracellular matrix stiffness: a material’s perspective. Current opinion in biotechnology. 2013;24(5):948–53. 23611564 10.1016/j.copbio.2013.03.020 PMC4037408

[29] Davidson CD, Jayco DKP, Matera DL, DePalma SJ, Hiraki HL, Wang WY, et al. Myofibroblast activation in synthetic fibrous matrices composed of dextran vinyl sulfone. Acta Biomater. 2020;105:78–86. doi: 10.1016/j.actbio.2020.01.009 31945504 PMC7369643

[30] d’Angelo M, Benedetti E, Tupone MG, Catanesi M, Castelli V, Antonosante A, et al. The Role of Stiffness in Cell Reprogramming: A Potential Role for Biomaterials in Inducing Tissue Regeneration. Cells. 2019;8(9):1036. doi: 10.3390/cells8091036 31491966 PMC6770247

[31] Moshayedi P, Costa L da F, Christ A, Lacour SP, Fawcett J, Guck J, et al. Mechanosensitivity of astrocytes on optimized polyacrylamide gels analyzed by quantitative morphometry. J Phys Condens Matter. 2010;22(19):194114. doi: 10.1088/0953-8984/22/19/194114 21386440

[32] Tang X, Zhang Y, Mao J, Wang Y, Zhang Z, Wang Z, et al. Effects of substrate stiffness on the viscoelasticity and migration of prostate cancer cells examined by atomic force microscopy. Beilstein J Nanotechnol. 2022;13:560–9. doi: 10.3762/bjnano.13.47 35860456 PMC9263554

[33] Tang X, Zhang Y, Mao J, Wang Y, Zhang Z, Wang Z, et al. Effects of substrate stiffness on the viscoelasticity and migration of prostate cancer cells by atomic force microscopy. Beilstein Archives. 2022;2022(1):13. 10.3762/bjnano.13.47 PMC9263554 35860456

[34] Daliri K, Pfannkuche K, Garipcan B. Effects of physicochemical properties of polyacrylamide (PAA) and (polydimethylsiloxane) PDMS on cardiac cell behavior. Soft Matter. 2021;17(5):1156–72. doi: 10.1039/d0sm01986k 33427281

[35] Sabass B, Gardel ML, Waterman CM, Schwarz US. High resolution traction force microscopy based on experimental and computational advances. Biophys J. 2008;94(1):207–20. doi: 10.1529/biophysj.107.113670 17827246 PMC2134850

[36] Kalli M, Poskus MD, Stylianopoulos T, Zervantonakis IK. Beyond matrix stiffness: targeting force-induced cancer drug resistance. Trends Cancer. 2023;9(11):937–54. doi: 10.1016/j.trecan.2023.07.006 37558577 PMC10592424

[37] Aydin HB, Ozcelikkale A, Acar A. Exploiting Matrix Stiffness to Overcome Drug Resistance. ACS Biomater Sci Eng. 2024;10(8):4682–700. doi: 10.1021/acsbiomaterials.4c00445 38967485 PMC11322920

[38] Mancini A, Gentile MT, Pentimalli F, Cortellino S, Grieco M, Giordano A. Frontiers in Oncology. 2024;14:1406644. 39015505 10.3389/fonc.2024.1406644 PMC11249764

[39] Nam A-Y, Joo SH, Khong QT, Park J, Lee NY, Lee S-O, et al. Deoxybouvardin targets EGFR, MET, and AKT signaling to suppress non-small cell lung cancer cells. Sci Rep. 2024;14(1):20820. doi: 10.1038/s41598-024-70823-7 39242647 PMC11379681

[40] Eker B, Meissner R, Bertsch A, Mehta K, Renaud P. Label-free recognition of drug resistance via impedimetric screening of breast cancer cells. PLoS One. 2013;8(3):e57423. doi: 10.1371/journal.pone.0057423 23483910 PMC3587579

[41] Binnig G, Quate C, Gerber C. Atomic force microscope. Phys Rev Lett. 1986;56(9):930–3. doi: 10.1103/PhysRevLett.56.930 10033323

[42] Lekka M, Laidler P, Gil D, Lekki J, Stachura Z, Hrynkiewicz AZ. Elasticity of normal and cancerous human bladder cells studied by scanning force microscopy. Eur Biophys J. 1999;28(4):312–6. doi: 10.1007/s002490050213 10394623

[43] Goldmann WH, Ezzell RM. Viscoelasticity in wild-type and vinculin-deficient (5.51) mouse F9 embryonic carcinoma cells examined by atomic force microscopy and rheology. Exp Cell Res. 1996;226(1):234–7. doi: 10.1006/excr.1996.0223 8660960

[44] Martens JC, Radmacher M. Softening of the actin cytoskeleton by inhibition of myosin II. Pflugers Arch. 2008;456(1):95–100. doi: 10.1007/s00424-007-0419-8 18231808

[45] Baker EL, Lu J, Yu D, Bonnecaze RT, Zaman MH. Cancer cell stiffness: integrated roles of three-dimensional matrix stiffness and transforming potential. Biophys J. 2010;99(7):2048–57. doi: 10.1016/j.bpj.2010.07.051 20923638 PMC3042573

[46] Corbin EA, Vite A, Peyster EG, Bhoopalam M, Brandimarto J, Wang X, et al. Tunable and Reversible Substrate Stiffness Reveals a Dynamic Mechanosensitivity of Cardiomyocytes. ACS Appl Mater Interfaces. 2019;11(23):20603–14. doi: 10.1021/acsami.9b02446 31074953 PMC13720955

[47] Wang X-P, Chen T-S, Sun L, Cai J-Y, Wu M-Q, Mok M. Live morphological analysis of taxol-induced cytoplasmic vacuolization [corrected] in human lung adenocarcinoma cells. Micron. 2008;39(8):1216–21. doi: 10.1016/j.micron.2008.04.007 18514532

[48] Au NPB, Fang Y, Xi N, Lai KWC, Ma CHE. Probing for chemotherapy-induced peripheral neuropathy in live dorsal root ganglion neurons with atomic force microscopy. Nanomedicine. 2014;10(6):1323–33. doi: 10.1016/j.nano.2014.03.002 24632247

[49] Kim KS, Cho CH, Park EK, Jung M-H, Yoon K-S, Park H-K. AFM-detected apoptotic changes in morphology and biophysical property caused by paclitaxel in Ishikawa and HeLa cells. PLoS One. 2012;7(1):e30066. doi: 10.1371/journal.pone.0030066 22272274 PMC3260205

[50] Lin C-CK, Yang C-H, Ju M-S. Cytotoxic and biomechanical effects of clinical dosing schemes of paclitaxel on neurons and cancer cells. Cancer Chemother Pharmacol. 2020;86(2):245–55. doi: 10.1007/s00280-020-04113-0 32683463

[51] Andolfi L, Bourkoula E, Migliorini E, Palma A, Pucer A, Skrap M, et al. Investigation of adhesion and mechanical properties of human glioma cells by single cell force spectroscopy and atomic force microscopy. PLoS One. 2014;9(11):e112582. doi: 10.1371/journal.pone.0112582 25390644 PMC4229222

[52] Milani P, Marilley M, Sanchez-Sevilla A, Imbert J, Vaillant C, Argoul F, et al. Mechanics of the IL2RA gene activation revealed by modeling and atomic force microscopy. PLoS One. 2011;6(4):e18811. doi: 10.1371/journal.pone.0018811 21533205 PMC3076448

[53] Rotsch C, Radmacher M. Drug-induced changes of cytoskeletal structure and mechanics in fibroblasts: an atomic force microscopy study. Biophys J. 2000;78(1):520–35. 10620315 10.1016/S0006-3495(00)76614-8 PMC1300659

[54] Ren J, Huang H, Liu Y, Zheng X, Zou Q. An Atomic Force Microscope Study Revealed Two Mechanisms in the Effect of Anticancer Drugs on Rate-Dependent Young’s Modulus of Human Prostate Cancer Cells. PLoS One. 2015;10(5):e0126107. doi: 10.1371/journal.pone.0126107 25932632 PMC4416805

[55] Suckow MA, Hiles MC. Use of Conditioned Extracellular Matrix as a Tissue-engineered Tumor Matrisome for Prostate Cancer and Melanoma Immunotherapy. Anticancer Res. 2023;43(1):335–41. doi: 10.21873/anticanres.16168 36585187

[56] Marr KD, Ignatenko NA, Warfel NA, Batai K, Cress AE, Pollock GR, et al. Digital image analysis using video microscopy of human-derived prostate cancer vs normal prostate organoids to assess migratory behavior on extracellular matrix proteins. Front Oncol. 2023;12:1083150. doi: 10.3389/fonc.2022.1083150 36727054 PMC9885251

[57] Treacy P-J, Martini A, Falagario UG, Ratnani P, Wajswol E, Beksac AT, et al. Association between Expression of Connective Tissue Genes and Prostate Cancer Growth and Progression. Int J Mol Sci. 2023;24(8):7520. doi: 10.3390/ijms24087520 37108678 PMC10139147

[58] Xu K, Huang Y, Wu M, Yin J, Wei P. 3D bioprinting of multi-cellular tumor microenvironment for prostate cancer metastasis. Biofabrication. 2023;15(3):10.1088/1758-5090/acd960. doi: 10.1088/1758-5090/acd960 37236173

[59] Kader A, Kaufmann JO, Mangarova DB, Moeckel J, Adams LC, Brangsch J, et al. Collagen-Specific Molecular Magnetic Resonance Imaging of Prostate Cancer. Int J Mol Sci. 2022;24(1):711. doi: 10.3390/ijms24010711 36614152 PMC9821004

[60] Feng D, Wang J, Shi X, Li D, Wei W, Han P. Membrane tension-mediated stiff and soft tumor subtypes closely associated with prognosis for prostate cancer patients. Eur J Med Res. 2023;28(1):172. doi: 10.1186/s40001-023-01132-4 37179366 PMC10182623

[61] Deng B, Zhao Z, Kong W, Han C, Shen X, Zhou C. Biological role of matrix stiffness in tumor growth and treatment. J Transl Med. 2022;20(1):540. doi: 10.1186/s12967-022-03768-y 36419159 PMC9682678

[62] Dean M, Fojo T, Bates S. Tumour stem cells and drug resistance. Nat Rev Cancer. 2005;5(4):275–84. doi: 10.1038/nrc1590 15803154

[63] Shen Y, Wang X, Lu J, Salfenmoser M, Wirsik NM, Schleussner N, et al. Reduction of Liver Metastasis Stiffness Improves Response to Bevacizumab in Metastatic Colorectal Cancer. Cancer Cell. 2020;37(6):800-817.e7. doi: 10.1016/j.ccell.2020.05.005 32516590

[64] Fan Y, Sun Q, Li X, Feng J, Ao Z, Li X, et al. Substrate Stiffness Modulates the Growth, Phenotype, and Chemoresistance of Ovarian Cancer Cells. Front Cell Dev Biol. 2021;9:718834. doi: 10.3389/fcell.2021.718834 34504843 PMC8421636

[65] Urbano RL, Furia C, Basehore S, Clyne AM. Stiff Substrates Increase Inflammation-Induced Endothelial Monolayer Tension and Permeability. Biophys J. 2017;113(3):645–55. doi: 10.1016/j.bpj.2017.06.033 28793219 PMC5550298

[66] Sun M, Chi G, Li P, Lv S, Xu J, Xu Z, et al. Effects of Matrix Stiffness on the Morphology, Adhesion, Proliferation and Osteogenic Differentiation of Mesenchymal Stem Cells. Int J Med Sci. 2018;15(3):257–68. doi: 10.7150/ijms.21620 29483817 PMC5820855

[67] Melica ME, La Regina G, Parri M, Peired AJ, Romagnani P, Lasagni L. Substrate Stiffness Modulates Renal Progenitor Cell Properties via a ROCK-Mediated Mechanotransduction Mechanism. Cells. 2019;8(12). 10.3390/cells8121561 PMC6953094 31816967

[68] Liu B, Kilpatrick JI, Lukasz B, Jarvis SP, McDonnell F, Wallace DM, et al. Increased Substrate Stiffness Elicits a Myofibroblastic Phenotype in Human Lamina Cribrosa Cells. Invest Ophthalmol Vis Sci. 2018;59(2):803–14. doi: 10.1167/iovs.17-22400 29392327

[69] Pandamooz S, Jafari A, Salehi MS, Jurek B, Ahmadiani A, Safari A, et al. Substrate stiffness affects the morphology and gene expression of epidermal neural crest stem cells in a short term culture. Biotechnol Bioeng. 2020;117(2):305–17. 31654402 10.1002/bit.27208

[70] Zonderland J, Wieringa P, Moroni L. A quantitative method to analyse F-actin distribution in cells. MethodsX. 2019;6:2562–9. doi: 10.1016/j.mex.2019.10.018 31763187 PMC6861648

[71] Prauzner-Bechcicki S, Raczkowska J, Madej E, Pabijan J, Lukes J, Sepitka J, et al. PDMS substrate stiffness affects the morphology and growth profiles of cancerous prostate and melanoma cells. J Mech Behav Biomed Mater. 2015;41:13–22. doi: 10.1016/j.jmbbm.2014.09.020 25460399

[72] Peng Y, Chen Z, He Y, Li P, Chen Y, Chen X, et al. Non-muscle myosin II isoforms orchestrate substrate stiffness sensing to promote cancer cell contractility and migration. Cancer Lett. 2022;524:245–58. doi: 10.1016/j.canlet.2021.10.030 34715250

[73] Zhang C, Tan Y, Feng J, Huang C, Liu B, Fan Z, et al. Exploration of the Effects of Substrate Stiffness on Biological Responses of Neural Cells and Their Mechanisms. ACS Omega. 2020;5(48):31115–25. doi: 10.1021/acsomega.0c04279 33324820 PMC7726759

[74] Zhao D, Xue C, Li Q, Liu M, Ma W, Zhou T, et al. Substrate stiffness regulated migration and angiogenesis potential of A549 cells and HUVECs. J Cell Physiol. 2018;233(4):3407–17. doi: 10.1002/jcp.26189 28940499

[75] Chen J, Backman LJ, Zhang W, Ling C, Danielson P. Regulation of Keratocyte Phenotype and Cell Behavior by Substrate Stiffness. ACS Biomater Sci Eng. 2020;6(9):5162–71. doi: 10.1021/acsbiomaterials.0c00510 33455266

[76] Halder G, Dupont S, Piccolo S. Transduction of mechanical and cytoskeletal cues by YAP and TAZ. Nat Rev Mol Cell Biol. 2012;13(9):591–600. doi: 10.1038/nrm3416 22895435

[77] Dupont S, Morsut L, Aragona M, Enzo E, Giulitti S, Cordenonsi M, et al. Role of YAP/TAZ in mechanotransduction. Nature. 2011;474(7350):179–83. doi: 10.1038/nature10137 21654799

[78] Ridley AJ. Rho family proteins: coordinating cell responses. Trends Cell Biol. 2001;11(12):471–7. doi: 10.1016/s0962-8924(01)02153-5 11719051

[79] Jaffe AB, Hall A. Rho GTPases: biochemistry and biology. Annu Rev Cell Dev Biol. 2005;21:247–69. doi: 10.1146/annurev.cellbio.21.020604.150721 16212495

[80] Pollard TD, Cooper JA. Actin, a central player in cell shape and movement. Science. 2009;326(5957):1208–12. doi: 10.1126/science.1175862 19965462 PMC3677050

[81] Aubert G, Lansdorp PM. Telomeres and aging. Physiol Rev. 2008;88(2):557–79. doi: 10.1152/physrev.00026.2007 18391173

[82] Starr DA, Fridolfsson HN. Interactions between nuclei and the cytoskeleton are mediated by SUN-KASH nuclear-envelope bridges. Annu Rev Cell Dev Biol. 2010;26:421–44. doi: 10.1146/annurev-cellbio-100109-104037 20507227 PMC4053175

[83] Lombardi ML, Lammerding J. Altered mechanical properties of the nucleus in disease. Methods Cell Biol. 2010;98:121–41. doi: 10.1016/S0091-679X(10)98006-0 20816233

[84] Kouzarides T. Chromatin modifications and their function. Cell. 2007;128(4):693–705. doi: 10.1016/j.cell.2007.02.005 17320507

[85] Berger SL. The complex language of chromatin regulation during transcription. Nature. 2007;447(7143):407–12. doi: 10.1038/nature05915 17522673

[86] Libring S, Solorio L. Cancer mechanobiology: Interaction of biomaterials with cancer cells. Biomaterials for cancer therapeutics. Elsevier. 2020;445–70.

[87] Gargalionis AN, Papavassiliou KA, Papavassiliou AG. Targeting the YAP/TAZ mechanotransducers in solid tumour therapeutics. J Cell Mol Med. 2023;27(13):1911–4. doi: 10.1111/jcmm.17794 37226849 PMC10315712

[88] Rubí-Sans G, Nyga A, Mateos-Timoneda MA, Engel E. Substrate stiffness-dependent activation of Hippo pathway in cancer associated fibroblasts. Biomater Adv. 2025;166:214061. doi: 10.1016/j.bioadv.2024.214061 39406156

[89] Gargalionis AN, Basdra EK, Papavassiliou AG. Mechanosignalling in tumour progression. J Cell Mol Med. 2018;22(2):704–5. 29134745 10.1111/jcmm.13452 PMC5783856

[90] Liu Z, Wang L, Xu H, Du Q, Li L, Wang L, et al. Heterogeneous Responses to Mechanical Force of Prostate Cancer Cells Inducing Different Metastasis Patterns. Adv Sci (Weinh). 2020;7(15):1903583. doi: 10.1002/advs.201903583 32775149 PMC7404165

[91] Lee HJ, Ewere A, Diaz MF, Wenzel PL. TAZ responds to fluid shear stress to regulate the cell cycle. Cell Cycle. 2018;17(2):147–53. 29143545 10.1080/15384101.2017.1404209 PMC5884395

