# Supplementary material for: Mechanochemical interactions in cancer cells: The role of substrate stiffness in cell behavior and drug response
Source: PLoS One. 2026 Jan 7;21(1):e0327874. doi: 10.1371/journal.pone.0327874 (PMC12779079; doi:10.1371/journal.pone.0327874)
Supplement: S1 File — Three samples are tested for each elastic modulus, (b) schematic of atomic force microscopy-based elastic modulus measurement of cells; A cantilever applies force to the cells, and a detector measures the resulting deflection, (c) the force-deflection curve as an example of the AFM outputs. (DOCX) [file pone.0327874.s001.docx]

In the present study, a tensile testing machine was used to measure the elastic modulus of the substrates, while an AFM was employed to evaluate the mechanical properties of the cells. Fig S1a illustrates the apparatus used to assess the mechanical properties of the fabricated substrates through pressure and tensile testing. Samples were placed in a cylindrical mold with a diameter and height of 1 inch and tested using a universal testing machine. Tensile tests were performed under controlled conditions with a strain rate of 20 mm/min and a preload of 10 N. For each substrate formulation, three independent samples were tested to ensure reproducibility and statistical reliability. The elastic modulus was calculated from the linear region of the stress–strain curve obtained during testing.

| (a) | |
| --- | --- |
| 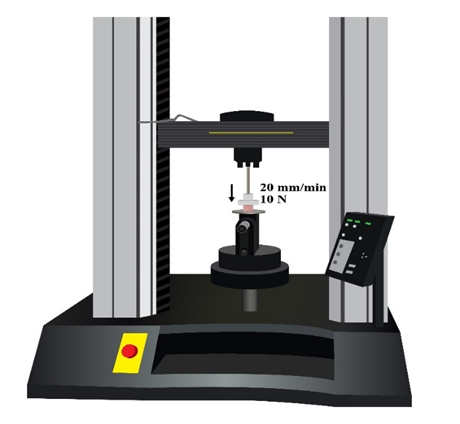 | |
| (b) | (c) |
| 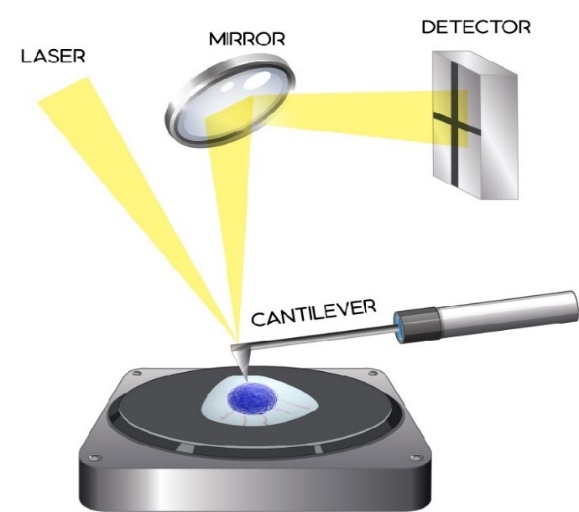 | 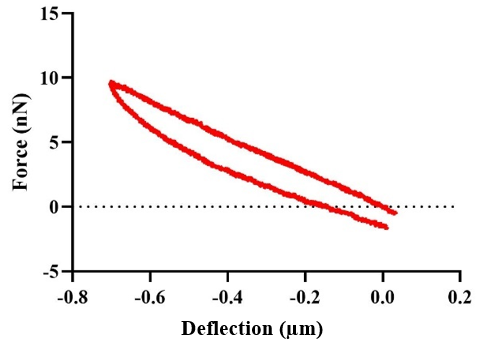 |

**Fig S1. (a) Tensile testing on the substrates; the samples are tested in a cylindrical mold with diameter and height of 1 inch under 20 mm per minute strain and 10 N load. Three samples are tested for each elastic modulus, (b) schematic of atomic force microscopy-based elastic modulus measurement of cells; A cantilever applies force to the cells, and a detector measures the resulting deflection, (c) the force-deflection curve as an example of the AFM outputs.**

The methodology employed for quantifying the mechanical properties of live cells using AFM is also illustrated in Fig S1. Panel (b) depicts a schematic representation of the AFM setup. In this configuration, a microfabricated cantilever with a sharp tip is brought into contact with the cell surface. As the cantilever indents the cell, the resulting deflection is detected via a laser beam reflected onto a position-sensitive photodetector. The measured deflection is directly related to the force applied by the cantilever tip, enabling high-resolution, nanoscale assessment of cell stiffness. Panel (c) presents a representative force–distance curve acquired during AFM indentation. This curve characterizes the mechanical response of a cell to applied force. By fitting the approach portion of the curve using the Hertz contact model, the apparent Young’s modulus of the cell can be quantitatively extracted. These measurements provide critical insights into the biomechanical behavior of cells and their mechanosensitive responses to varying substrate stiffness.
